# Measuring sidewalk distances using Google Earth

**DOI:** 10.1186/1471-2288-12-39

**Published:** 2012-03-29

**Authors:** Ian Janssen, Andrei Rosu

**Affiliations:** 1 School of Kinesiology and Health Studies, Queen's University, 28 Division St., Kingston, ON, K7L 3N6, Canada; 2 Department of Community Health and Epidemiology, Queen's University, Kingston, Canada

## Abstract

**Background:**

Physical activity is an important determinant of health. Walking is the most common physical activity performed by adults and the presence of sidewalks along roads is a determinant of walking. Geographic information systems (GIS) can be used to measure sidewalks; however, GIS sidewalk data are difficult to access. The purpose of this study was to present a new GIS method for measuring the distance and coverage of sidewalks along roadways.

**Methods:**

The new method contains three stages. Stage 1 involves calculating the distance of all road segments within the region of interest (e.g., neighborhood), extracting geospatial information on these road segments, and saving this information as a Google Earth file. This stage was performed in ArcGIS software. Stage 2 involves opening the extracted road segment geospatial data in Google Earth, visually examining road segments to see if they contain sidewalks, and deleting road segments without sidewalks. Stage 3 involves importing the modified road geospatial data into ArcGIS and calculating the length of road segments with sidewalks. The new method was tested in 315 sites across Canada. Each site consisted of a one km radius circular buffer surrounding a school.

**Results:**

A detailed, step-by-step protocol is provided in the paper. The length of road segments with sidewalks in the testing sites ranged from 0.00 to 55.05 km (median 16.20 km). When expressed relative to the length of all road segments, the length of road segments with sidewalks ranged from 0% to 100% (median 53%). By comparison to urban testing sites, rural sites had shorter sidewalk lengths and a smaller proportion of the roads had sidewalk coverage.

**Conclusion:**

This study provides a new GIS protocol that researchers can use to measure the distance and coverage of sidewalks along roadways.

## Background

Walking is the most common physical activity [1]. People who walk more in their leisure-time or as a form of active transportation have lower risks for developing type 2 diabetes, cardiovascular disease, certain cancers, and premature mortality [2-4]. Active transportation also reduces automobile use and greenhouse gas emissions, thereby improving air quality and decreasing acute and chronic respiratory illnesses within the population [5,6].

In an effort to increase the physical activity and active transportation rates in the population, researchers have been studying the determinants of these behaviors. One such determinant is the walkability of neighborhoods and communities [5,7-11]. Factors affecting walkability include the speed limits and connectivity of roads and the presence of sidewalks. Roads with high speed limits and roads that are poorly connected can make it unsafe and inefficient (i.e., long travel distances) for people to walk on and use for active transportation [7-9]. Sidewalks provide a pedestrian right-of-way on the roadside, and not surprisingly, traffic accidents involving pedestrians are far less common on roads that contain sidewalks [12,13]. Furthermore, the distance of sidewalks within a neighborhood, as well as the proportion of roads with sidewalk coverage, predicts active transportation. For instance, the active transportation to school literature has demonstrates that walking to school is positively correlated with the sidewalk coverage along roads (e.g., percentage of roads in the school neighborhood with sidewalks) [14,15]. Furthermore, the construction of new sidewalks along existing roadways contributed to increased active transportation to school rates within several communities that participated in California's safe routes to school intervention [16].

The walkability features of roads are typically measured using geographic information systems (GIS). GIS data on roads is widely available in most, if not all, industrialized countries. For instance, the CanMap^® ^RouteLogistics database (DMTI Spatial Inc., Markham, ON) provides geospatial information on public roads across Canada. GIS can also be used to measure the length and connectivity of sidewalks; however, GIS sidewalk data are far less common than road data and can be difficult and in some cases impossible to access. In Canada, for example, GIS sidewalk data are only available in larger cities, and this information is difficult for researchers to access as many cities do not share their GIS data. Furthermore, the GIS sidewalk data are collected and maintained in a different manner in different cities. Collectively, these issues make it challenging to conduct sidewalk-based research studies in smaller municipalities and in larger geographical areas (e.g., state/province or national level).

The purpose of this technical advance was to present a new GIS method for measuring sidewalk distances that can be used across regions and even entire countries. The method relies on an existing GIS road database and freely accessible Google Earth^® ^mapping software. We also tested this method in 315 locations across Canada to illustrate how it can be used. Although this method was developed and tested in Canada using a Canadian road network database, it could be easily extracted to other countries and databases.

## Methods

### Development of method

The new method for measuring sidewalk distances contains three stages, and each of these stages contains several steps. *Stage 1 *involves calculating the distance of all road segments within the region of interest (including road segments without sidewalks), extracting geospatial information on these road segments, and saving this information as a Google Earth file. A road segment refers to a length of road having similar features, such as a city block. Within the boundaries of the cities and towns in our testing sites the road segments were 131 meters in length on average, and in rural areas they were 1871 meters in length on average. This stage was performed using CanMap RouteLogistics (DMTI Spatial Inc., Markham, ON) in ArcGIS software version 9.3 (Esri, Redlands, CA). CanMap RouteLogistics provides geospatial data on roads across Canada. *Stage 2 *involves opening the extracted road segment geospatial data in Google Earth, visually examining each road segment in aerial and street view images to see if sidewalks are present on either sides of the road for each road segment, and manually deleting any road segments that do not have a sidewalk on at least one side from the road segment geospatial data. *Stage 3 *involves importing the modified road segment geospatial data back into ArcGIS - this modified data represents the road segments that have sidewalks - and calculating the distance of road segments in the region of interest that contain sidewalks. More details for each of the three stages are contained in the Results section.

### Testing of method

We tested the newly developed method in 315 locations across Canada from October 2010 to March 2011. One km radius circular buffers (area of 3.14 km^2^) surrounding 315 schools that participated in the Canadian version of the 2009/2010 Health Behaviour in School-Aged Children Survey (HBSC) acted as the testing sites. The HBSC sampled schools from 8 of 10 Canadian provinces (New Brunswick and Prince Edward Island were not included) and all 3 Canadian territories. A single stage cluster sampling approach was used to obtain schools. The sample of schools is representative by school board type (public or separate), language of instruction, urban/rural geography, and regional geography.

Within each 1 km circular buffer testing site we calculated the total distance of road segments with sidewalks; hereafter we refer to this as sidewalk distance. We also calculated the proportion of road segments that had a sidewalk (total distance of road segments with a sidewalk ÷ total distance of all road segments ×100); hereafter we refer to this as sidewalk coverage. Descriptive information on these two measures was provided for all 315 testing sites and according to urban/rural geography. For the urban/rural comparisons, testing sites were classified as rural areas (< 10,000 people; N = 78), small cities (10,000 - 99,999 people; N = 116), or metropolitan areas (≥ 100,000 people; N = 121) based on the population of the municipality where the testing site was located.

## Results

### Development of method

A detailed step-by-step description of the newly developed protocol for measuring sidewalk distances is provided below. An example from one of the testing sites has been included within the step-by-step description to provide an illustration of how the new method works.

#### Stage 1 - exporting road network file from ArcGIS

*Step 1: *Adding GIS layers

• Open ArcMap software.

• Selecting "Add data".

• Open up the map of the area where you will be measuring the sidewalks. In the example we opened up a map of Canada.

• Add the road network layer for the area where you will be measuring sidewalks. In the example the road network layer for the specific testing site was obtained from CanMap RouteLogistics and was located in the DMTI Spatial Data folder.

• Add a layer that contains the water bodies. This layer is not required and this step is optional.

• Add the layer for the point of the specific location where you want to measure sidewalk distances. In the example we selected a point shapefile for the street address of one of the school testing sites. This file was called "School_Address".

*Step 2: *Create the area or buffer around the specific point (from *Step 1*) where you want to measure the distance of sidewalks

**Figure 1:**
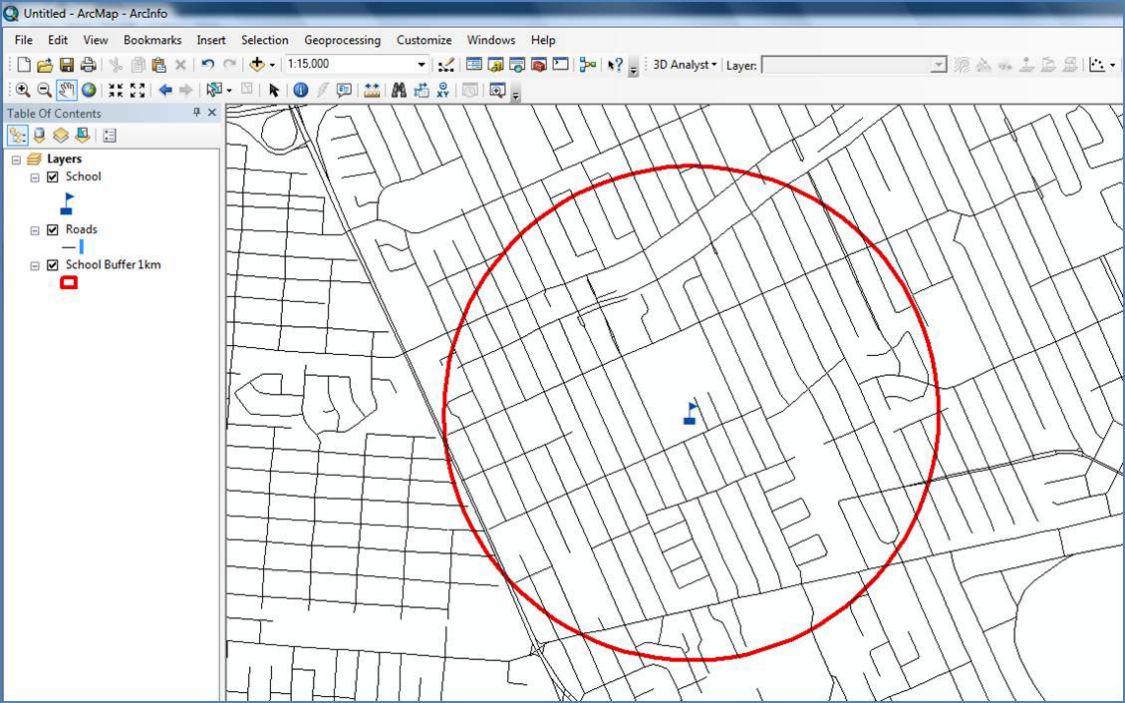
**Computer screen shot of the road network layer for a 1 km radius circular buffer testing site obtained within ArcGIS**. The red circle represents the buffer, the black lines are the roads, and the blue flag in the center of the circle is a school.

• Select the "ArcToolbox" icon, navigate to "Analysis Tools", then to "Proximity", then to "Buffer".

• On the "Input Features" of the pop-up-box, type in the file name (or select the point layer from the option box) for the point shapefile that was selected in *Step 1*. This file was called "School_Address" in the example.

• In the "Output Features Class" select the directory where you want to export the shapefile layer for the buffer, and give the shapefile a name. This shapefile was called "School_Buffer" in the example.

• In this example, which was based on a 1 km shapefile buffer, under "Distance [value or field]" we selected "Linear unit", we typed in "1" in the text box below "Linear unit", and we selected "kilometers" in the menu beside "Linear unit". These specifications can be modified depending on the type, size, and distance units of the buffer being used.

• Select "OK" at the bottom of the pop-up-box.

• An illustration of what the computer screen looked like at the end of *Step 2 *for the example testing site is shown in Figure 1.

*Step 3 (optional): *Change the colour, symbol type and/or width of layers

• Click the left mouse button while the cursor is located on the layer symbols if you want to change the visual features of these symbols.

*Step 4: *Extract road network geospatial data

• Select the "ArcToolbox" icon, navigate to "Analysis Tools", then to "Overlay" then to "Intersect".

• In the pop-up-box select the buffer that was created in *Step 2*. This was called "School_Buffer" in the example.

• Select the road network layer that was added in *Step 1*.

• Under "Output Feature Class" select the directory where you want to save the new file, and then give this file a name. In the example, we called this file "Extracted_Roads".

• Select "OK" on the bottom of the pop-up-box.

*Step 5: *Save the extracted road network and the buffer layer as a KML file.

**Figure 2:**
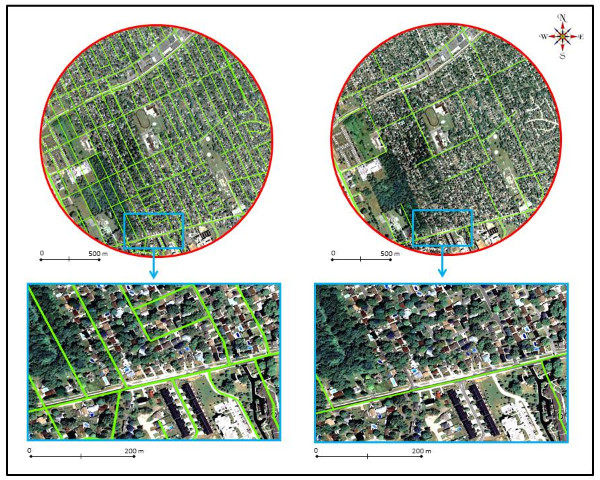
**Overview of the GIS sidewalk measures for a 1 km radius circular buffer testing site**. The top left panel shows the 1 km circular buffer testing site, and all of the road segments are highlighted with the green lines. The top right panel shows the same 1 km circular buffer testing as in the top left panel; however, only the road segments that have a sidewalk are highlighted with the green lines. The bottom left and bottom right panels of the figure show a blow-up of a section of the 1 km circular buffer testing site. All road segments (bottom left panel) and those road segments with sidewalks (bottom right panel) are highlighted with the green lines.

• Remove (by checking-off) the point shape file layer, which was called "School_Address" in our example, and the original road network layer.

• Double click on the road network symbol (under Layers table of contents) and change the color and width of the road segment lines. We suggest you select a bright green color and a width value of 3, which were used in our illustrative example (Figure 2).

• Double click on the buffer layer symbol (under the Layer table of contents) and select a bright colour that is different than the road network color. We suggest red, which was used in our example.

• Select the "ArcToolbox" icon and navigate to "Conversion Tools", then to "To KML", then to "Layer To KML".

• In the pop-up box, under "Layer" type in the file name for the extracted road network from *Step 4 *("Extracted_Roads" in the example)

• Under "Output File" select the directory where you want to save the KML file.

• Under "Layout Output Scale" type in "1", which is the scale.

• Select "OK" on the pop-up-box.

• Select the "ArcToolbox" icon and navigate to "Conversion Tools", then to "To KML", then to "Layer To KML".

• In the pop-up box, under "Layer" type in the file name for the newly created buffer layer from *Step 2 *("School_Buffer" in the example).

• Under "Output File" select the directory where you want to save the KML file.

• Under "Layout Output Scale" type in "1".

• Select "OK" on the pop-up-box.

#### Stage 2 - deleting road segments without sidewalks in Google earth

*Step 1: *Open up the road network and buffer layers in Google Earth.

• Open Google Earth. This software can be downloaded from http://www.google.com/earth/index.html.

• In the main table of contents select "File", navigate to the folder where the KML files were saved in *Step 5 *of *Stage 1*, and double click with the left mouse button on both files. These files were called "Extracted_Roads" and "School_Buffer" in the example.

• The road network and buffer layers should now appear in Google Earth. An illustration of these two layers for the example test site is shown in the top left panel of Figure 2.

Step 2: Deleting road segments without sidewalks

**Figure 3:**
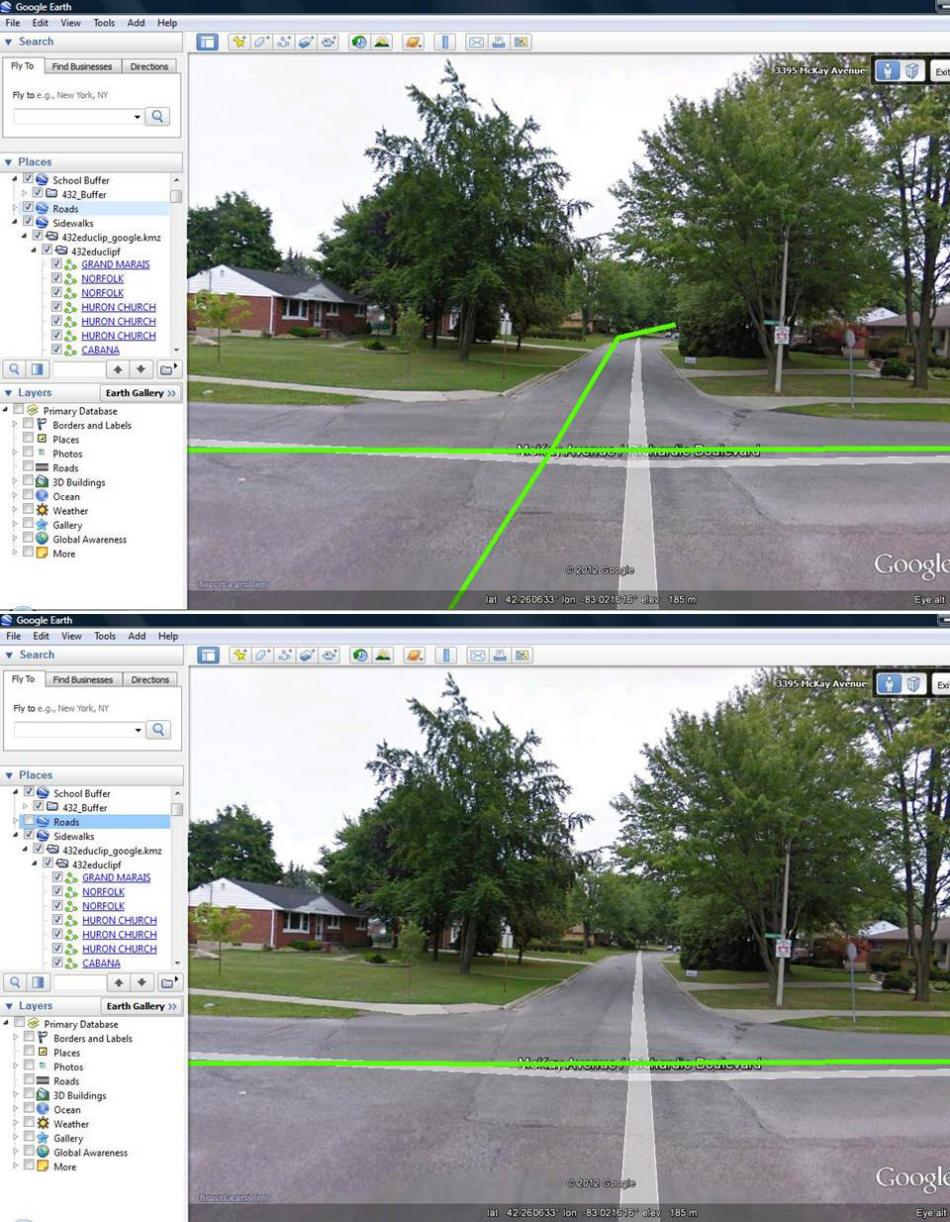
**Computer screen shot of road segments within a testing site obtained at the street view level within Google Earth**. In the top panel all of the visible road segments are highlighted with green lines. In the bottom panel only the road segments that are covered by a sidewalk are highlighted with green lines. Thus, the top and bottom figures represent the "before" and "after" of the step where road segments without sidewalks were deleted.

• On the "Places" table of contents click on the "+" button beside the name of the road network layer that was opened in *Step 1 *("Extracted_Roads" in the example). This will open a list of the road segments that are located within the road shapefile buffer.

• Double click with the left mouse button on the first road segment that appears in the list. This will take Google Earth to the location of the selected road, which will appear on the image in the main part of the screen. An image of what this may look on a computer screen is shown in Figure 3.

• Visually inspect the segment to see if that road contains sidewalks on either or both sides. This process can be facilitated by zooming, panning or by using the street view option.

• If the road segment does not have a sidewalk on at least one side, delete that road segment by clicking on that road segment and selecting "delete". If the road segment has a sidewalk on one or both sides, do nothing.

• Repeat *Step 2 *for all the road segments in the shapefile buffer and delete all road segments that do not contain sidewalks. The top right panel in Figure 2 displays the road network pattern in the example buffer after all road segments without sidewalks have been deleted. Notice the differences between the green road network pattern in the top left and top right panels. These differences reflect the roads without sidewalks. See Figure 3 for an illustration of these differences.

*Step 3: *Save the modified road network (i.e., road segments with sidewalks) as a Google Earth file.

• Select the road network layer in the "Places" table of contents (called "Extracted_Roads" in the example) and select "Save Place As". In the example we called this new file "Roads_with_Sidewalks".

#### Stage 3 - calculating the length of sidewalks along roads

Step 1

• *O*pen ArcMap

• Select the "ET Geowizards Tool" icon (*Note*: you may need to install ET Geowizards. This program can be downloaded at http://www.ian-ko.com/.

• In the pop-up-box select the "Import/Export" option from the left menu, and then the "Import from Google Earth" option.

• Select "Go" on the pop-up-box.

• In the new pop-up-box, in the "Select Google Earth file" text box, type in or select the Google Earth road network file that was saved in *Step 5 *of *Stage 2*. This file was called "Roads_with_Sidewalks" in the example.

• In the "Specific output PDGB or folder" text box, specify the output folder.

• Select "Add layers to the Map" and then "Finish".

• Close the two pop-up-boxes.

*Step 2: *Provide the data frame with the proper map projection in order to accurately calculate the length of the roads with sidewalks.

• Select the data frame name (Layers) and choose "Properties".

• In the pop-up-box select the "Predefined" folder, and within this folder select "Projected Coordinate Systems" then the "UTM" folder then the "NAD 1983" folder and then the appropriate Universal Transverse Mercator (UTM) geographic zone. Note that if you are calculating sidewalk distances outside of North America you should select "WGS 84" instead of "NAD 83". In the example, "UTM NAD83 Zone 17 N" was selected as the sidewalks were being measured for a testing site that was located within this UTM zone. Sixteen different UTM zones were used in our national study of 315 Canadian schools.

• Select "Apply" and then "OK".

Step 3:

• In the "Layer" table of contents, select the imported road layer (this was called "Roads_with_Sidewalks" in the example) and then "Attribute Table".

• In the pop-up-box select "Options" and then "Add field".

• In the new pop-up-box type in a name for the new field. This new filed was called "Sidewalk_Length" in the example.

• Under "Type" select the "Double" option. For the "Field Properties" leave "Precision" as 0 and "Scale" as 0.

• Select "OK".

*Step 4*: Calculate the distance of each road segment that has a sidewalk.

• In the pop-up-box select the new field that was created in *Step 3 *(this field was called "Sidewalk_Length" in the example) and then the "Calculate Geometry" option.

• Under "Property" select "Length".

• Under "Coordinate System" select the "Use coordinate system of the data frame" option.

• Under "Units" select the unit of measure (e.g., kilometers, meters) you want the sidewalk distance to be measured in.

• Select "OK".

*Step 5: *Calculate the distance of road segments containing sidewalks for the entire buffer.

**Figure 4:**
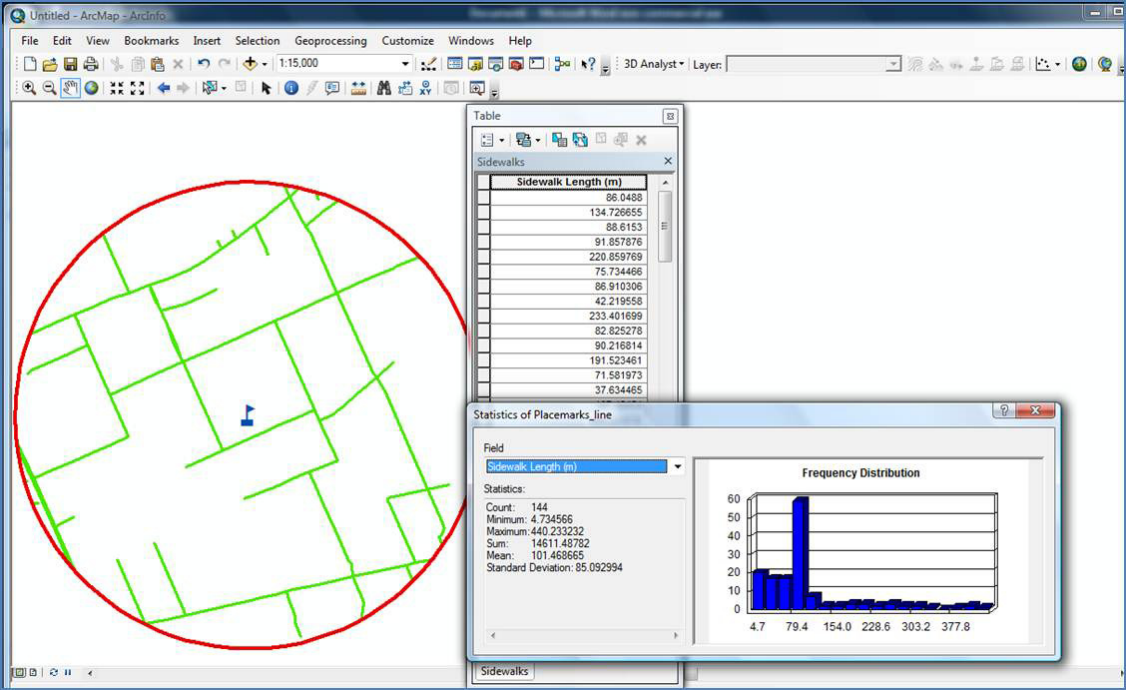
**Computer screen shot of the road network layer with sidewalk coverage for a 1 km radius circular buffer testing site obtained within ArcGIS and the summary table with the sidewalk length details (right side)**.

• In the pop-up-box select the new field that was created in *Step 3 *(this field was called "Sidewalk_Length" in the example) and select "Statistics".

• Within the new pop-up-box the following statistics will be provided in a summary table: count (which is the number of road segments with a sidewalk in buffer), minimum (which is the length of the shortest road segment in the buffer that has a sidewalk), maximum (which is length of the longest road segment in the buffer that has a sidewalk), sum (which is total length of road segments in the buffer that have a sidewalk), mean (which is the average length of road segments in the buffer that have a sidewalk), and standard deviation (which is standard deviation of the mean).

• An illustration of what the computer screen looked like at the end of *Step 5 *for the example testing site is shown in Figure 4.

*Step 6*: Record sidewalk distance information

• Either manually record the data from the summary table or copy-and-paste it into another file type such as Excel.

### Testing of method

The time required to obtain the sidewalk distance measures within the 1 km circular buffer testing sites varied considerably. Some of the testing sites that were located in rural areas only contained a few roads and were completed in approximately 5 minutes. Some of the testing sites that were located in metropolitan areas contained dense networks of roads and took over 30 minutes to complete.

Table 1 contains the median, interquartile range, minimum, and maximum values for the sidewalk distance measures that were obtained for the 315 testing sites where Google Street View data was available (*note*: Google Street View was not available for 121 of the total 436 school sampled for the HBSC). The distance of road segments with sidewalks in the testing sites ranged from 0 to 55.05 km, with a median of 16.20 km. When expressed relative to the distance of all road segments, the sidewalk coverage ranged from 0% to 100%, with a median of 52.8%. There were clear urban-rural gradients in these sidewalk measures such that the rural testing sites had shorter sidewalk distances and a smaller proportion of their roads had sidewalk coverage.

**Table 1:** Descriptive information on distance of sidewalks collected in the 1 km radius circular buffer testing sites across Canada

Testing Site	Lowest	25^th ^percentile	50^th ^percentile	75^th ^percentile	Highest
*Sidewalks distance in 1 km buffer (km)*					

Total (N = 315)	0	8.06	16.20	28.61	55.05

Rural (N = 78)	0	1.84	4.24	10.40	26.14

Small cities (N = 116)	0	10.23	15.32	24.28	55.05

Metropolitan (N = 121)	0	20.63	27.73	33.76	51.53

Percentage of total road distance in 1 km buffer that had a sidewalk (%)*					

Total (N = 315)	0	31.6	52.8	78.1	100

Rural (N = 78)	0	11.5	23.7	43.1	90.1

Small cities (N = 116)	0	39.9	52.8	73.2	98.3

Metropolitan (N = 121)	0	54.1	76.7	86.4	100

* calculated as length of road segments with a sidewalk/length of all road segments ×100

## Discussion

The purpose of this technical advance was to present the protocol of a new GIS method for measuring the distance and coverage of sidewalks along roadways. The new sidewalk measurement protocol could be used in a variety of research, urban planning, and public health settings. The protocol could be used in descriptive research studies where sidewalk characteristics are being compared across different neighborhoods, communities, etc. It could be used in etiological research studies that aim to characterize the relationship between the presence and length of sidewalks with walking behaviors. Municipalities that do not have geospatial sidewalk data could use this protocol to create such information. Public health and educational authorities could use the sidewalk measures obtained in this protocol, in combination with road data, to identify safe routes for school-aged children to use when walking to school (i.e., all road segments used on travel route must have a sidewalk).

While obtaining the sidewalk measures in our testing sites we encounter three common issues. We discuss those issues here as well as the solutions we used to overcome them. First, for private sections of a neighborhood and gated communities images were not always available at the street level. In these instances, we were required to zoom out to the aerial view level. Second, some of the roads that were ≥ 4 lanes wide only had street view images for one side of the road. In these instances we navigated to the nearest intersection and did a 360 degree panoramic view to see if a view of the other side of the road was available, and if it was not, we zoomed out to the aerial view level. Third, in densely populated urban cores there were often trucks, buses, construction sites, etc. that blocked the view of the side of the road. In these instances we navigated forward or backward along the road at the street view level until we could see around the object(s), or if need be, zoomed out to the aerial view level.

The sidewalk measurement protocol developed in this study could be expanded in a number of ways. First, in addition to measuring the distance of sidewalks, it would be possible to measure the connectivity of sidewalks, in a similar manner to how road (street) connectivity measures are obtained. In fact, because 48% of the roads in the test sites of this national study did not have sidewalk coverage, it may be more appropriate to measure sidewalk connectivity rather than road connectivity in active transportation focused studies. The sidewalk connectivity measures could rely on the same type of indicators that are used to measure road connectivity such as the density of intersections per unit area, the percentage of intersections that are 3- or 4-way intersections, and the number of dead ends [7]. Researchers could take the sidewalk shapefiles saved at the end of Stage 2 in the protocol (i.e., road network that contains sidewalks) and obtain the connectivity measures on these files instead of the complete road network.

It may also be possible to integrate qualitative measurements of the sidewalks into the protocol, such as the sidewalk surface (e.g., pavement, asphalt, brick, etc.), sidewalk condition (e.g., excellent, in need of some repairs, in need of major repairs), and proximity of the sidewalks to the roads (e.g., directly beside road, boulevard separating road and sidewalk). Thus, rather than measuring the distance of all sidewalks, these distance measures could be broken down by qualitative features (e.g., distance of sidewalks in poor, fair, and good condition). Previous built environment research on parks has demonstrated that Google Earth can be used to obtain valid qualitative data [17]. It would be possible to integrate this type of information into Stage 2 of the sidewalk measurement protocol. More specifically, each of the road segments with a sidewalk could be coded based on its qualitative features (i.e., rather than deleting a road segment; code it a different color based on its qualitative features). Thus, in Stage 3 the distances of sidewalks that meet different characteristics could be calculated.

A key advantage of the newly developed method is having the ability to measure sidewalk distances and coverage in a consistent manner in several different municipalities. This will allow researchers who are interested in these types of studies to conduct regional, national, and even international studies. A key limitation of the newly developed method is that Google Earth does not provide street view and high resolution aerial images in all areas. In particular, these types of images are not available in many rural and remote areas. Another main limitation is the timing of when the Google Earth aerial and street view images were obtained relative to when the research study is completed. For example, in our city (Kingston, Ontario) the aerial images for Google Earth were obtained in 2004 and the street view images were obtained in 2009. If we were to conduct a Kingston-based study in 2012, some of the sidewalks in the city would have changed since 2004 and 2009, particularly in newly developed areas. Thus, as with most GIS measures of the built environment, the sidewalk measurement protocol would have a limited utility in newly developed areas.

## Conclusion

The measurement of built environment constructs is becoming an increasingly important component of public health research. This study provides a new measurement protocol that researchers can use to measure the distance and coverage of sidewalks along roadways. It is hoped that the use of this protocol in future studies will lead to an improved understanding of the walking environment and the determinants of walking in different areas.

## Competing interests

The authors declare that they have no competing interests.

## Authors' contributions

AR developed the sidewalk measurement protocol, obtained the sidewalk measures for many of the testing sites, and revised the manuscript for important intellectual content. IJ came up with the study idea, was responsible for writing the manuscript, and completing the statistical analysis. Both authors approve the version that has been submitted.

## Pre-publication history

The pre-publication history for this paper can be accessed here:

http://www.biomedcentral.com/1471-2288/12/39/prepub
